# Suppression of NF-κB Reduces Myocardial No-Reflow

**DOI:** 10.1371/journal.pone.0047306

**Published:** 2012-10-09

**Authors:** Min Zeng, Hongbing Yan, Yi Chen, Han-jun Zhao, Yuan Lv, Cheng Liu, Peng Zhou, Bo Zhao

**Affiliations:** 1 Department of Cardiology, Beijing Anzhen Hospital, the Capital Medical University, Beijing, China; 2 National Center for Cardiovascular Diseases, Cardiovascular Institute and Fuwai Hospital, Peking Union Medical College and Chinese Academy of Medical Sciences, Beijing, China; 3 The People's Hospital of Hainan Province, Haikou, China; National Institutes of Health, United States of America

## Abstract

No-reflow phenomenon is a risk factor which severely compromises the benefits of coronary revascularization in patients with acute myocardial infarction. Inflammatory response, as an essential component of cardiac ischemia/reperfusion (I/R) injury, has been suggested to contribute to the myocardial no-reflow. Since nuclear factor kappa B (NF-κB) is a key mediator of inflammation, we reasoned that inhibition of NF-κB might reduce the extent of no-reflow. To test this hypothesis, the left circumflex coronary arteries of New Zealand white male rabbits were ligated for 1.5 h, followed by reperfusion for 1 h to induce I/R injury. Pretreatment of the rabbits with a specific NF-κB inhibitor, pyrrolidine dithiocarbamate (PDTC), significantly attenuated neutrophil infiltration in the no-reflow area as well as the expansion of no-reflow. These beneficial effects were associated with a marked reduction in the serum levels of myocardial induced I/R tumor necrosis factor-α (TNF-α), intercellular adhesion molecule-1 (ICAM-1), and CXCL16. Consistently, simulative I/R culture of human umbilical vein endothelial cells (HUVECs) resulted in an increase of TNF-α, ICAM-1 and CXCL16, and all of these changes were significantly suppressed by pretreatment of the cells with PDTC or with siRNA-mediated p65 knockdown. Our data thus suggest that inhibition of NF-κB may reduce I/R-associated myocardial no-reflow through reduction of myocardial inflammation.

## Introduction

No-reflow (NR) phenomenon is the failure of blood to reperfuse an ischemic area after the physical obstruction has been removed or bypassed by percutaneous coronary intervention, thrombolysis and coronary artery bypass grafting [1]. The incidence and extent of NR strongly predict adverse clinical outcomes including persistent contractile dysfunction of left ventricular, malignant arrhythmias and cardiac death [2]. Although the mechanisms of NR are not completely understood, existing evidences from both clinic setting [3] and animal NR model [4] support the involvement of ischemia/reperfusion (I/R) injury, vasospasm, neutrophils plugging, and endothelial swelling. Recently, it was reported that patients with higher C-reactive protein levels and white cell count tend to suffer from NR [5], [6]. This suggests the possibility that inflammatory cells and proinflammatory cytokines cells may directly mediate the occurrence and development of NR. However, the underlying mechanisms responsible for neutrophils infiltration and increased chemokine expression in NR have not been fully elucidated.

Nuclear factor kappa-B (NF-κB) is a key mediator of inflammation. P50/p65 heterodimer, which is one of the most avidly forming dimmers and is the major complex in most cells, is commonly referred specifically and hereinafter as NF-κB. p65, in particular, was indicated to be involved in the inflammation of myocardial I/R injury [7]. Bound with its inhibitory IKB protein, NF-κB is presented as an inactive complex in cytoplasm till extracellular signals activate IkB kinase which targets IKB for degradation and releases NF-κB. Then the unmasked NF-κB translocates into the nucleus to activate the pivot transcriptions of target genes and mediates immune and inflammatory responses by manipulating many inflammatory cytokines, adhesion molecules and chemokines. Activation of NF-κB constitutes the central part of many cardiovascular inflammatory diseases. Inflammatory response is one of the main mechanisms of I/R injury [3]. As the earliest initiation factor of inflammation, NF-κB has been proven to play a key role in myocardial I/R process [8]. However, whether NF-κB is involved in the pathogenesis of myocardial NR remains unknown. Based on our previous observation that NF-κB is markedly activated in the cardiac NR area (Data not shown), we speculate that NF-κB may promote NR through mediating inflammation in response to myocardial I/R. To this end, we utilized a specific NF-κB inhibitor, pyrrolidine dithiocarbamate (PDTC) in a rabbit myocardial I/R model and investigated the association between the levels of NF-κB, neutrophil infiltration, the expression of inflammatory cytokines and the extent of myocardial NR. Furthermore, we performed simulative I/R culture of human umbilical vein endothelial cells (HUVECs) to determine the expression levels of proinflammatory cytokines in the presence or absence of PDTC or specific p65 siRNA.

## Materials and Methods

### Ethics statement

All animals used in this study received humane care in compliance with the Guide for the Care and Use of Laboratory Animals, NIH Publication, 1996 edition, and all the protocols were approved by the Animal Subjects Committee of Capital Medical University, Beijing, China.

### Rabbit myocardial ischemia/reperfusion (I/R) procedures

New Zealand white male rabbits weighing 2.0–3.0 kg (aged 3–5 months) were anesthetized by sodium pentobarbital (30 mg/kg iv.) and the surgery was performed as following. In brief, after 1.5 h ligation of the left circumflex (LCX) coronary artery, the occlusion was released for 1 h (10 rabbits per group). Rabbits were treated with vehicle (saline) or PDTC (20 mg/kg) intravenously 0.5 h before the onset of I/R and therefore were divided into I/R and I/R+PDTC group accordingly. We checked PDTC of 20 mg/kg and 40 mg/kg in our preliminary study and 20 mg/kg PDTC was proven to be safe and efficient. Rabbits in Sham group underwent all the same surgical procedures except that the sutures, passing around the LCX, were not tied.

### Myocardial damage measurement

Myocardial damage was measured by using Evan's blue and Thioflavin S staining methods as previously described [9]. Briefly, 1 ml/kg of thioflavin S (6%, Sigma) was injected into the left atrium at the end of I/R procedure for the assessment of NR area. Thioflavin S is a specific endothelial fluorescent dye, by which fluorescent negative area of the heart was recognized as NR area. 1 min later, the LCX coronary artery was retied at the exact previous occlusion site followed by left atrium injection of 2 ml 4% Evan's blue (Alfa Aesar) to measure the ischemic risk area (AAR). The rabbits were then sacrificed using an overdose of sodium pentobarbital. The heart was quickly removed, sliced into five transverse sections, weighted, observed and photographed under ultraviolet light (254 nm). The left ventricular area (LV), AAR (defined as not stained by the blue dye), non-ischemic area (N, stained by the blue dye) and NR area for all slices were calculated by computer-assisted planimetry using Imagepro-plus software. Samples from three areas (N, AAR and NR) of LV cardiac tissue were processed accordingly for subsequent analysis.

### Reagents

Antibodies for NF-κB p65 and β-Actin were from Santa Cruz Biotechnology (Santa Cruz, CA); antibody for p-NF-κB p65 was from Cell Signaling Technology (Beverly, MA). PDTC was purchased from Sigma.

### Cell culture

Human umbilical vein endothelial cells (HUVECs) were obtained from ScienCell Research Laboratories (Carlsbad, CA, USA) and cultured in Endothelial Cell Medium (ECM) supplemented with endothelial cell growth supplement (ECGS), 5% fetal bovine serum (FBS) and penicillin/streptomycin (P/S) solution (ScienCell, Carlsbad, CA, USA). For experiments, cells of passage 4–6 were grown until confluence. PDTC was added 1 h prior to the exposure of HUVECs to normal condition or simulative I/R at the concentrations of 0.1 mM, 0.25 mM and 0.5 mM. Typan blue staining confirmed no toxicity effects of PDTC in this setting.

### HUVECs I/R protocol

Simulative I/R was initiated by incubation of HUVECs for 30 min in an “ischemic buffer” containing 118 mM NaCl, 24 mM NaHCO3, 1.0 mM NaH2PO4,2.5 mM CaCl2, 1.2 mM MgCl2, 20 mM sodium lactate, 16 mM KCl, 10 mM 2-deoxyglucose (pH adjusted to 6.2) [7], followed by “reperfusion” for 4 h. “Reperfusion” was accomplished by replacing the ischemic buffer with normal medium and culturing cells under normoxic condition.

### *In vitro* siRNA transfection

Pre-designed and validated siRNA specific for NF-κB p65, and control non-targeting siRNA were purchased from Santa cruz (sc-29410). HUVECs were plated to reach 30–50% confluence on the day of transfection, and 20 nM siRNA duplexes were introduced with Lipofectamine RNAiMAX (Invitrogen).

### Real Time RT-PCR

The quantitatively RT-PCR method has been described by us previously [10]. Total RNA was extracted from HUVECs using the trizol reagent (Invitrogen), and quantitative reverse transcription PCR was performed using the iScript™ cDNA Synthesis kit (BIO-RAD). 18S rRNA served as an endogenous control. We used a SYBR Premix Ex Taq (TaKaRa) and iQ5 Real-time PCR detection system (BioRad) for real-time quantification. Primer sequences were 5′-CCTGGAGCAGGCTATCAGTC-3′ and 5′-ATCTTGAGCTCGGCAGTGTT-3′ for NF-κB p65, and 5′-CAGAGGTTGAACCCCACAGT-3′ and 5′-CCTCTGGCTTCGTCAGAATC-3′ for ICAM-1, 5′-CCTGTGAGGAGGACGAACAT-3′ and 5′-AGGCCCCAGTTTGAATTCTT-3′ for TNF-α, 5′-CTCCTGGCCATCATCTTCAT-3′ and 5′-AAGCTTCCATTCTTGGCTCA-3′ for CXCL16, 5′-CGGCTACCACATCCAAGGAA-3′ and 5′-GCTGGAATTACCGCGGCT-3′ for 18S rRNA.

### ELISA

Blood samples were withdrawn from carotid artery of the rabbits at different time points (i.e. 0, 45, 100 and 150 minutes after ischemia) during the process of myocardial I/R. TNF-α, ICAM-1 and CXCL16 serum levels were analyzed using rabbit ELISA kits (XiTang Biotechnology Co., Ltd, Shanghai, China) according to manufacturers' instructions. The culture media of HUVECs with various treatments were also collected in *in vitro* study and subjected to human specific ELISA Kits (Quantikine; R&D Systems, Minneapolis, MN) to detect the supernatant levels of TNF-α, ICAM-1 and CXCL16 by a microplate reader set to 450 nm.

### Western blotting

Total HUVECs lysates from cell culture were separated in 10%SDS–PAGE and transferred onto polyvinylidene difluoride (PDVF) membrane (Millipore) and analysed by Western blotting as mentioned earlier [10]. The isolation of nuclear and cytophasmic protein from rabbit heart tissue and HUVECs was achieved by using NE-PER Nuclear and Cytoplasmic Extaction Kit (Thermo Scientific) according to the manufacturers's instructions.

### Immunohistochemistry

Heart samples were fixed in 4% paraformaldehyde solution and embedded in paraffin. Serial 5-µm sections were incubated with antibodies against NF-κB p65 (sc-8008, 1∶50, Santa Cruz) followed by counterstaining with hematoxylin. Images were viewed and captured blindly by two observers using of a Nikon Labophot 2 microscope equipped with a Sony CCD-Iris/RGB colour video camera attached to a computerized imaging system and analyzed by ImagePro Plus 3.0.

### Electron microscopy

Electron microscopy analysis was performed as described previously. Samples were taken from the centre part of NR area in sham, I/R and I/R+PDTC groups (n = 3 for each group) after myocardial I/R in the presence or absence of PDTC treatment 0.5 h ahead. Ultra-thin sections (1 mm^3^) were fixed for 30 min with ice-cold 2.5% glutaraldehyde in 0.1 M cacodylate buffer, embedded in Epon, and processed for transmission electron microscopy by standard procedures in a blinded fashion at ×6,000 magnification.

### Myeloperoxidase measurement (MPO)

MPO is an enzyme present in leukocytes and is a marker of leukocytes infiltration into myocardium. An MPO enzyme immunosorbent assay kit (JianCheng Bioengineering Institute, Nanjing, China) was used to determine MPO levels. Briefly, tissues from each area of the hearts were snapped frozen in liquid nitrogen until they were analyzed. A 5% (wt/vol) homogenate of each area of heart tissue in ice-cold PBS containing leupeptin (1 µg/ml), pepstatin A (1 µg/ml), and antipain (50 µg/ml) was prepared and centrifuged, and the supernatants were used for assay of MPO activity according to the manufactory's instructions and determined spectrophotometrically at 460 nm.

### Statistical analysis

The data were expressed as means±SD. Differences between the control and experimental groups were analyzed using Strudent's t-test. The data from more than two groups were evaluated by one-way ANOVA followed by the Newman-Keuls test. *P* value<0.05 is considered statistically significant.

## Results

### Suppression of NF-κB attenuates neutrophil infiltration in the NR area following I/R injury

**Figure 1:**
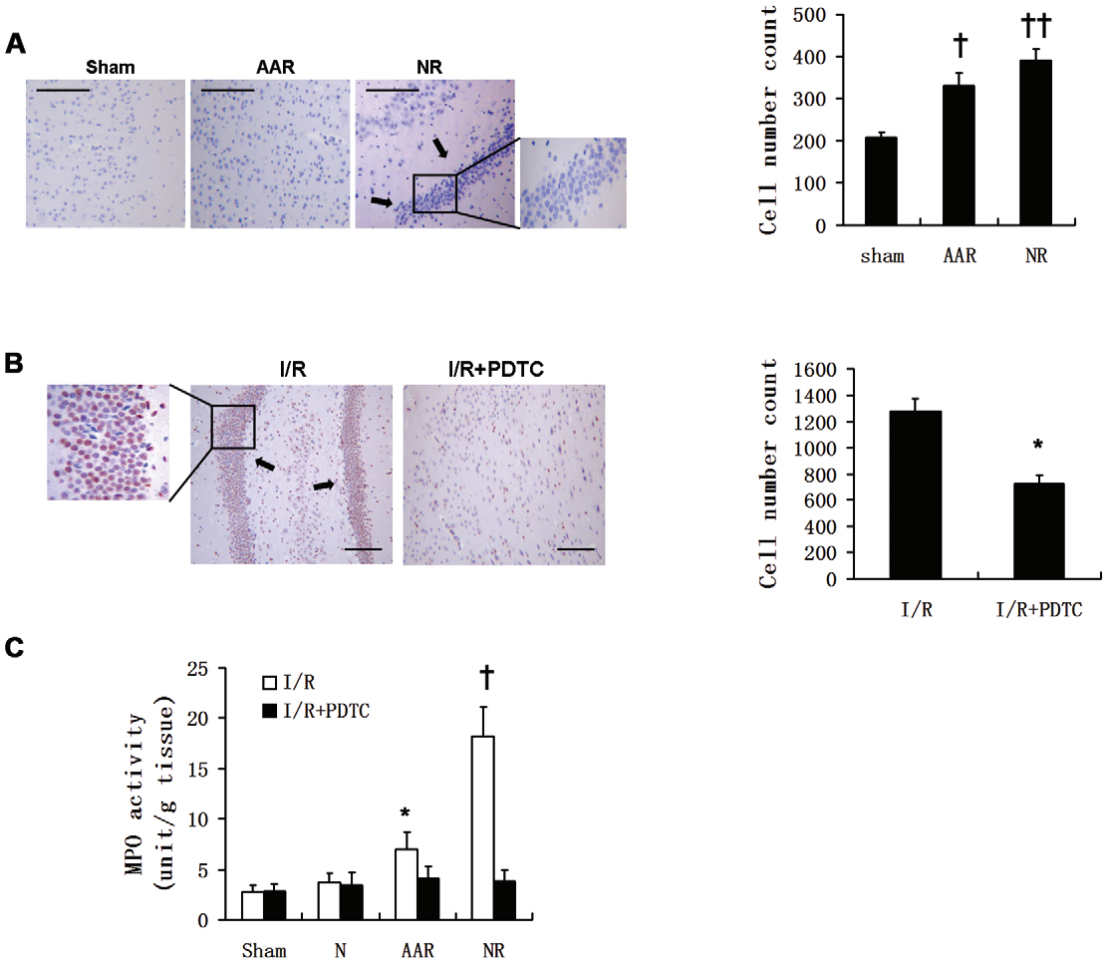
Myocardial I/R-induced infiltration of neutrophils in the NR area is attenuated by PDTC treatment. Male New Zealand white rabbits were treated with PDTC and 0.5 h later, underwent ischemia/reperfusion (I/R) by LCX coronary artery ligation for 1.5 h followed by reperfusion for 1 h. Sham group served as control. Non-ischemia (N), area at risk (AAR) and no-reflow (NR) were determined by Evan's blue and Thioflavin S staining. (**A**) H&E staining showing normal myocardium (Sham group), AAR and neutrophil infiltration in the NR area of I/R group (*left panels*). Images in each field were viewed and captured blindly by two observers and cell numbers in each field were quantified (*right panel*). ^†^P<0.01; ^††^P<0.001. (**B**) Immunohistochemical staining of p65 in the NR area of I/R group vs. I/R+PDTC group (*left panels*) and cell numbers quantification in NR area of I/R and I/R+PDTC group (*right panel*). *P<0.05. (**C**) Myocardiac myeloperoxidase (MPO) levels in the N, AAR and NR areas of sham, I/R, and I/R+PDTC groups. *P<0.05, vs. sham or AAR of I/R+PDTC; ^†^P<0.01, vs. sham or NR of I/R+PDTC. Scale bar, 30 µm. Results are expressed as mean±SD.

Myocardial I/R was induced by ligation of the left circumflex coronary artery of New Zealand white male rabbits for 1.5 h followed by reperfusion for 1 h. The non-ischemic (N), area at risk (AAR), and NR areas were identified by thioflavin S (a fluorescent dye specific for endothelial cells) and Evan's blue staining to facilitate histological and biochemical investigations for each individual area. The round, polymorphonuclear cells accumulated abundantly in strip-shape in the NR area of myocardial samples from I/R group, but absent in the normal myocardium (sham group) (**Left panel,** **Fig. 1A**). Quantification of cell numbers in each field was performed by viewing, capturing the images and analyzing the images with ImagePro Plus 3.0. software. The results revealed cell numbers in AAR and NR area increased by 1.6-fold (P<0.01) and 1.9-fold (P<0.001), respectively, compared to their counterparts in sham group (**Right panel,** **Fig. 1A**). Immunohistochemical staining of p65 revealed that most of the nuclei of these intensively infiltrated round-shaped cells were p65 positive (**Left panel,** **Fig. 1B**). Notably, striped gathering of polymorphonuclear cells was morphologically barely observed by pre-treatment of animals with PDTC, a specific NF-κB inhibitor. Cell numbers in NR area were accordingly decreased by 43% with PDTC treatment (P<0.05) (**Right panel,** **Fig. 1B**). To verify these accumulated polymorphonuclear cells were activated neutrophils, we measured myeloperoxidase (MPO) in each area of the hearts. MPO is most abundantly presented in neutrophil granulocytes and released into extracellular space via degranulation after the activation of neutrophils. Thus, not only MPO serves as a marker for neutrophils but also its level stands for the function and activity of neutrophils. Our results demonstrated that myocardial MPO levels in N, AAR and NR areas of I/R group were 1.3-fold, 2.5-fold (P<0.05), and 6.5-fold (P<0.01) of the sham group, respectively. Moreover, the MPO levels of N, AAR and NR areas in I/R+PDTC group were reduced by 7%, 41% (P<0.05), and 79% (P<0.01) respectively, as compared to those in the I/R group (**Fig. 1C**). Taken together, our data demonstrate that active neutrophils accumulate in the NR area of hearts after I/R, and inhibition of NF-κB with PDTC significantly reduces the sequestration and activity of neutrophils in this area.

### NF-κB inhibition decreases serum TNF-α, ICAM-1 and CXCL16 and ameliorates myocardial damage

**Figure 2:**
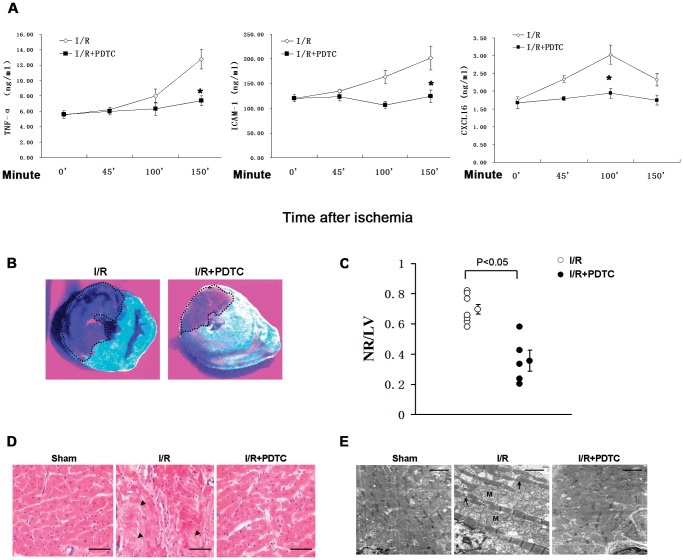
I/R increases serum levels of TNF-α, ICAM-1 and CXCL16 and aggravates cardiac damage through NF-κB. (**A**) Serum levels of TNF-α, ICAM-1 and CXCL16 at indicated time points after ischemia in I/R and I/R+PDTC group were detected by ELISA. n = 6. *, P<0.05. (**B**) Representative Thioflavin S staining showing the gross appearance of heart sections of I/R and I/R+PDTC groups. The NR area is negative for fluorescence as indicated by the broken lines. (**C**) Statistical analyses of NR/LV area ratio in I/R and I/R+PDTC groups. (I/R, n = 7; I/R+PDTC, n = 5). (**D**–**E**) Representative light (**D**) and electron (**E**) micrographs of NR area from sham, I/R, and I/R+ PDTC groups. **D**, H&E staining indicated severe interstitial edema and myocardial fibers disarrangement in NR area of I/R group (*middle panel*) in contrast to sham (*left panel*) or I/R+PDTC (*right panel*) group. Bar = 100 µm. **E**, Cardiomyocytes in NR area of I/R group (*middle panel*) displayed snapped myofibrils (arrows) and mitochondrial swelling (M), which was barely observed in sham group (*left panel*) or NR area of I/R+PDTC group (*right panel*). Figures are representative images of at least three different heart samples. Scale bar = 0.6 µm.

Because cytokines recruit and activate neutrophil granulocytes during the process of inflammation, we then sought to determine whether and when proinflammatory cytokines were upregulated in the setting of NR and whether the upregulation was mediated by NF-κB. In the rabbits in which NR developed following myocardial I/R, serum levels of TNF-α and ICAM-1 were markedly increased (**Fig. 2**). Levels of TNF-α at I/R 100 min (i.e., 100 min after ischemia) and 150 min were increased by 1.4-fold (P<0.05) and 2.3-fold (P<0.01), respectively, as compared to I/R 0 min (i.e., time right before ischemia) (upper left panel, **Fig. 2A**). Similarly, ICAM-1 levels at I/R 100 min and 150 min were elevated by 1.4-fold (P<0.05) and 1.7-fold (P<0.05), respectively, as compared to I/R 0 minute (upper middle panel, **Fig. 2A**). Pretreatment of rabbits with PDTC significantly suppressed I/R-induced elevations of TNF-α and ICAM-1 by 42% (P<0.05) and 38% (P<0.05), respectively, at I/R 150 minutes as compared to I/R group. Recently, CXCL16, a new chemokine was identified and added to the growing family of chemokines. Serum CXCL16 level was upregulated at early phrase of reperfusion (at I/R 100 minutes) with a maximal induction of 1.7-fold as compared to I/R 0 minute (P<0.05) (upper right panel, **Fig. 2A**). In contrast, serum CXCL16 level was lower by 36% when PDTC was used (P<0.05, I/R+PDTC vs. I/R) at the same time point. The I/R-induced NR area was evaluated by Thioflavin S staining (**Fig. 2B**), and demonstrated a 49% reduction with PDTC treatment (P<0.05) (**Fig. 2C**). The damage of reperfusion in NR area was revealed by H&E staining as the sections from NR area of I/R group exhibited severe interstitial edema and disarrangement of myocardial fibers in comparison with that of sham group (**Fig. 2D**). Furthermore, electron microscopy (EM) detected the ultrastructural damages of cardiomyocytes such as detached and snapped myofibrils, intramitochondrial blebs and mitochondrial edema with loss of cristae in NR area of I/R group (**Fig. 2E**). In contrast, all these damages displayed in NR area of I/R group were rescued in the same area of I/R+PDTC group.

### NF-κB inhibitor suppresses simulative I/R-induced expression of TNF-α, ICAM-1 and CXCL16

**Figure 3:**
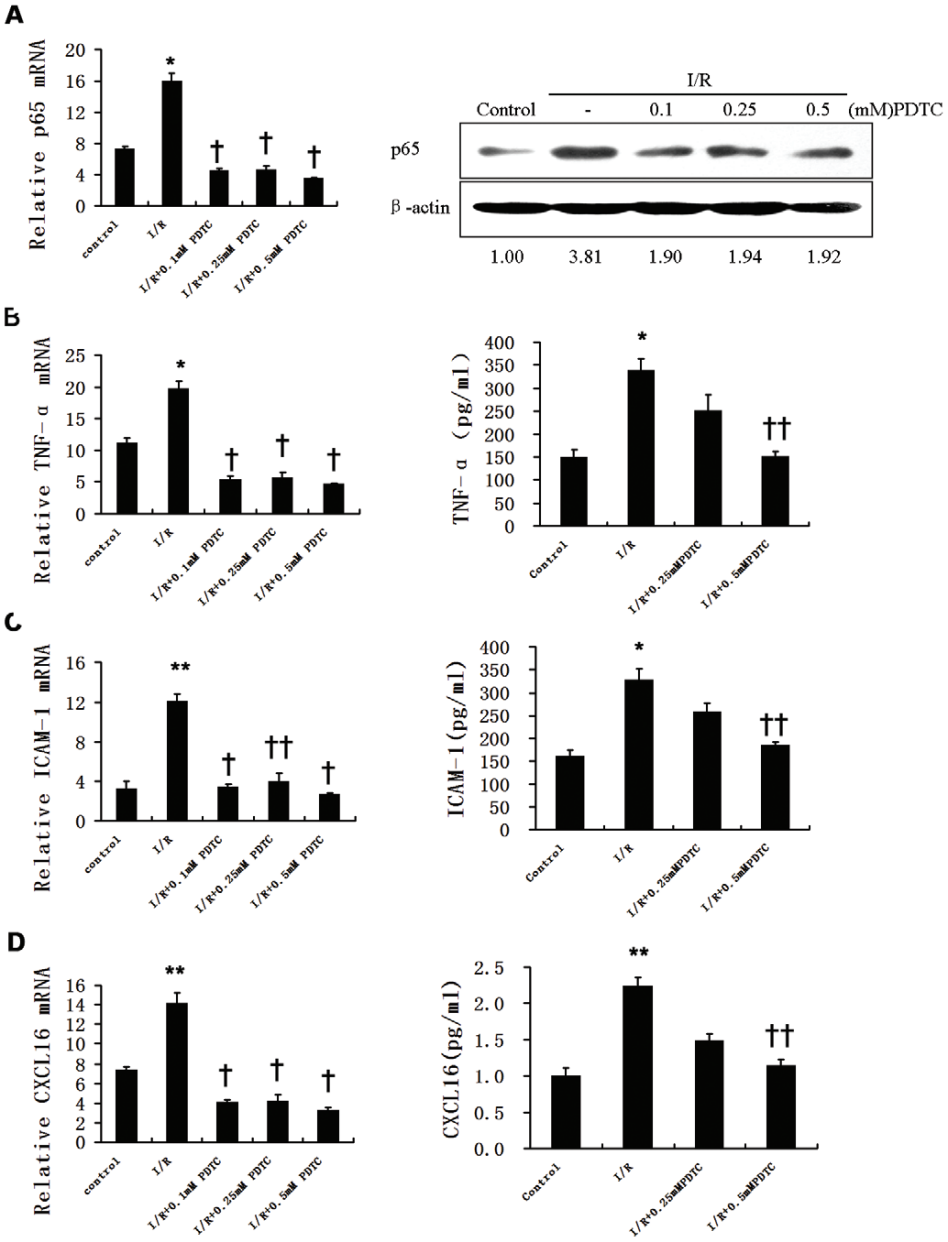
PDTC suppresses simulative I/R-induced mRNA and protein levels of TNF-α, ICAM-1 and CXCL16 in HUVECs. HUVECs were treated with incremental concentrations of PDTC for 1 h, then cultured in normal culture media (control) or in “ischemic buffer” for 30 min, followed by switching to normal media for 4 h (i.e., simulative I/R). Then, quantitative RT-PCR (*left panel*) and western blotting (*right panel*) were performed first to check the mRNA and protein levels of inflammatory markers p65 (**A**), followed by quantitative RT-PCR and ELISA to quantify cellular mRNA expression (*left panels*) and supernatant protein levels (*right panels*), respectively, of TNF-α (**B**), ICAM-1 (**C**) and CXCL16 (**D**). β-actin was used as a loading control. Densitometry values for p65 are expressed as fold change compared with control values normalized to 1. The results were obtained from three independent experiments carried out in duplicates and expressed as fold changes relative to the levels in control cells cultured in normal culture media. Data are presented as mean±SD. *, P<0.05 vs. control; **, P<0.01 vs. control; ^†^, P<0.01 vs. I/R; ^††^, P<0.05 vs. I/R.

To investigate the mechanisms underlying I/R injury-induced expression of proinflammatory cytokines and the repressor role of PDTC, we developed an in vitro simulative I/R culture system, in which cells were firstly treated with PDTC or vehicle for 1 h, then cultured in a well-defined “ischemic buffer” or normal medium (control) for 30 min, followed by switching to normal media for 4 h. Then various inflammatory mediators were analyzed quantitatively with quantitative RT-PCR, Western blotting, and ELISA. Because endothelial cells play a pivotal role in preserving vascular integrity and are very sensitive to the changes of flow environment as the very inner layer of the vessel, we chose to use HUVECs, which also appear to be one of the primary targets for I/R, for these studies. Simulative I/R treatment led to an increase in p65 mRNA level by 2.19-fold (P<0.05). Pretreatment of cells with 0.1 mM, 0.25 mM and 0.5 mM of PDTC significantly decreased the I/R-induced p65 mRNA expression by 72%, 71% and 78%, respectively (P<0.01) (**Left panel,** **Fig. 3A**). Accordingly, I/R induced p65 protein level by 3.81-fold (P<0.01), which was decreased by 0.5 mM PDTC by 49.6% (P<0.05) as quantified by densitometry and normalized to the values of β-actin (loading control) (**Right panel,** **Fig. 3A**). Simulative I/R significantly increased the levels of cellular mRNA and supernatant protein, respectively, by 1.77-fold and 2.27-fold for TNF-α (P<0.05), 3.69-fold (P<0.01) and 2.03-fold (P<0.05) for ICAM-1, 1.92-fold and 2.24-fold for CXCL16 (P<0.01), as compared to controls. Treatment of cells with 0.5 mM PDTC markedly decreased the simulative I/R-induced mRNA level of TNF-α to a maximal extent by 77% (P<0.01) and suppressed supernatant TNF-α level by 55% (P<0.05) in a dose dependent manner (with a maximal inhibition at 55%, P<0.05) (**Fig. 3B**). Similarly, mRNA and protein levels of ICAM-1 were significantly decreased by 78% (P<0.01) and 44% (P<0.05), respectively, with 0.5 mM PDTC treatment (**Fig. 3C**). Consistently, 0.5 mM PDTC suppressed I/R-induced mRNA and protein levels of CXCL16 to a maximal extent by 77% (P<0.01) and 49% (P<0.05), respectively (**Fig. 3D**).

### Depletion of NF-κB decreases simulative I/R-induced expression of TNF-α, ICAM-1 and CXCL16

**Figure 4:**
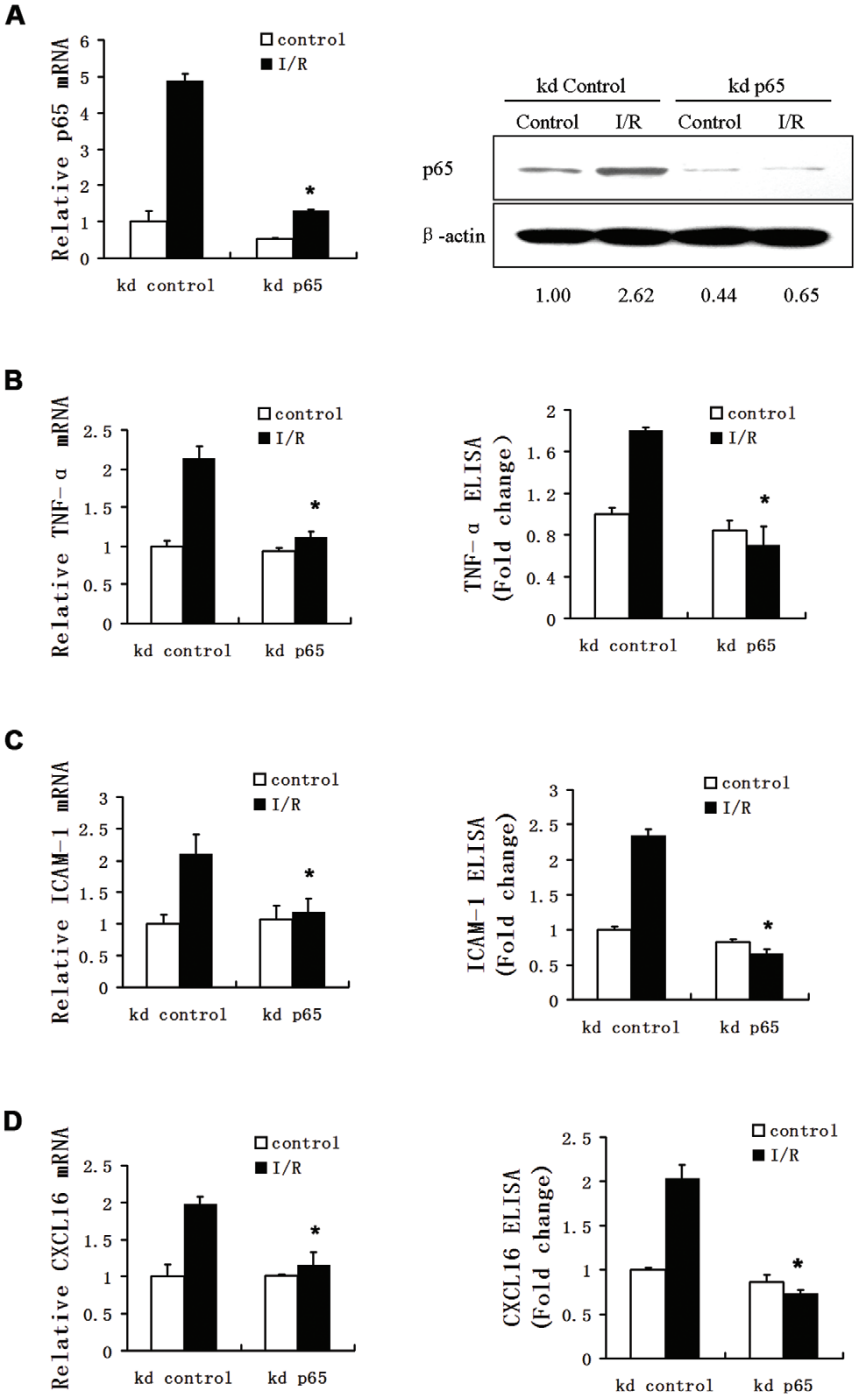
Knockdown of p65 attenuates the simulative I/R-induced elevations in mRNA and protein levels of TNF-α, ICAM-1 and CXCL16 in HUVECs. HUVECs were transfected with a p65-specific siRNA or a control non-targeting siRNA and 44 later, then exposed to normal culture media (control) or “ischemic buffer” for 30 min, followed by switching to normal media for 4 h. (A) Harvested cells were subjected to real-time RT-PCR for p65 mRNA expression (*Left panel*) or Western blotting for p65 protein levels (*Right panel*).β-actin was used as a loading control. Densitometry values for p65 are expressed as fold change compared with control values normalized to 1. (B–D) Relative cellular mRNA expression and supernatant protein levels of TNF-α (B), ICAM-1 (C) and CXCL16 (D) were evaluated by quantitative RT-PCR (Left panel) and ELISA (Right panel), respectively. The results were obtained from three independent experiments carried out in duplicates. The values are expressed as fold change relative to that in HUVECs transfected with non-targeting siRNA and cultured in normal media. Data are expressed as mean±SD. *, P<0.05 vs. simulative I/R without siRNA treatment. kd, knockdown.

We further confirm our observations by genetically knocking down p65 in HUVECs via RNA interference. Transfection of the cells with p65 siRNA decreased p65 mRNA level by 73% (P<0.05) as compared to transfection with control non-targeting siRNA (**Left panel,** **Fig. 4A**). Western blotting further confirmed the inhibitory effect of p65 siRNA on protein level. Transfection of 20 nM p65 siRNA under I/R condition reduced the protein level of p65 by 75.2% (P<0.01) (Right panel, **Fig. 4A**). Consequently, p65 siRNA transfection decreased I/R-induced mRNA and protein levels of TNF-α by 48% and 61%, respectively (P<0.05) (**Fig. 4B**), which was associated with a reduction in the mRNA and protein levels of ICAM-1 (by 44% and 72%, P<0.05) (**Fig. 4C**) and CXCL16 (by 42% and 64%, P<0.05) (**Fig. 4D**), respectively. Collectively, our data indicate that both NF-κB inhibitor PTDC and p65 knockdown significantly suppress simulative I/R-induced inflammatory cytokines TNF-α, ICAM-1 and CXCL16 and that NF-κB may play a central role in the inflammatory response to I/R injury.

### I/R promoted the activity of NF-κB in NR area of rabbit myocardium and HUVECs

**Figure 5:**
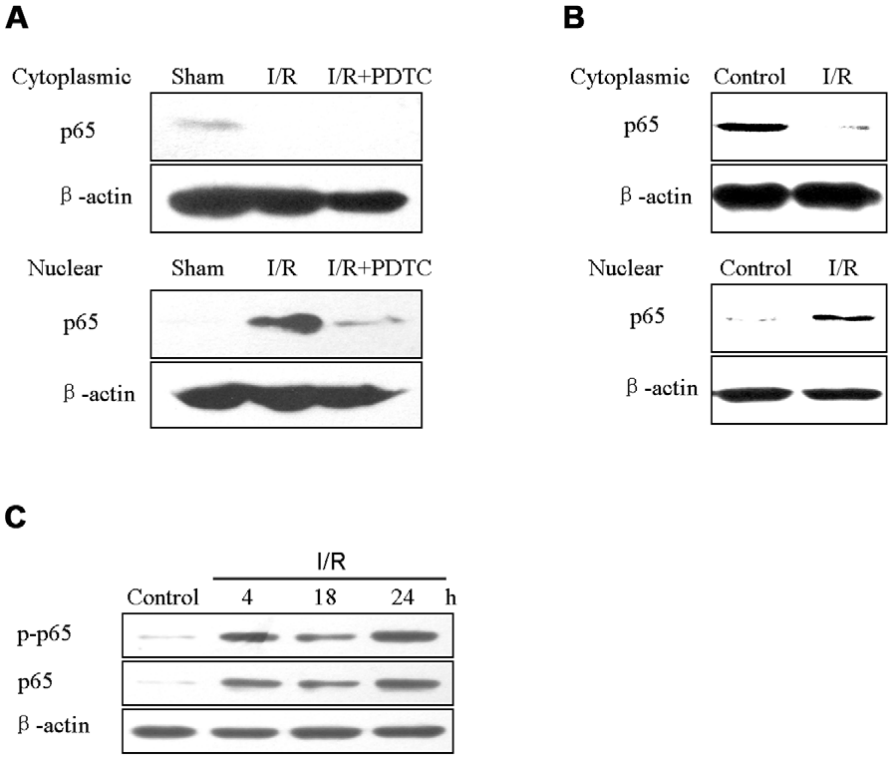
I/R induced nuclear translocation of NF-κB p65 in the NR area of rabbit heart and HUVEC cells. (**A**) Representative Western blots analysis of p65 in NR area of rabbit myocardial cytoplasmic and nuclear extracts from sham, I/R and I/R+PDTC groups. (**B**) HUVECs underwent simulative I/R for 4 h, then cytoplasmic and nuclear extracts of which were assessed by western blotting for p65. (**C**) HUVECs exposed to normal culture media (control) or 30 min of “ischemic buffer” followed by switching to normal media for 4 h, 18 h and 24 h. Cell lysates were then subjected to immunoblot for p-p65 (Ser536) and p65. β-actin served as internal control.

Nuclear translocation of p65 was determined by western blotting to confirm the activation of p65 after I/R. For *in vivo* study, nuclear protein of p65 in NR area from I/R group was dramatically elevated compared to sham group, which was quenched in I/R+PDTC group (**Fig. 5A**). We then sought to explore whether *in vitro* simulative I/R also activated p65 in HUVECs. Cytoplasmic and nuclear lysates of HUVECs were subjected to immunoblotting of p65 to determine nuclear translocation under control or simulative I/R circumstances. The results showed that simulative I/R indeed boosted the translocation of p65 into nuclei of HUVECs (**Fig. 5B**). I/R resulted in the increase of p65 protein level, which occurred within 4 h of reperfusion and persisted for at least 24 h. In accord with it, a reperfusion time-dependent activation of p65, as measured by phosphorylation at Ser536, was also observed in HUVECs after “ischemia” for 30 min (**Fig. 5C**).

## Discussion

NR phenomenon refers to that compromised microcirculation does not allow normal myocardial blood flow despite the removal of the coronary obstruction [11]. With the development of reperfusion strategies, it becomes more important to reduce the incidence of NR for a better clinic outcome. Myocardial I/R induces inflammatory response that contributes to NR, whereas the underlying mechanism remains unclear. The present study demonstrates that NF-κB, as a quick stress-reactive transcription factor, promotes the expression of proinflammatory cytokines and mediates the recruitment of neutrophils in the NR area to precipitate dysfunction of microcirculation *in vivo*. Furthermore, our *in vitro* studies confirm that NF-κB mediates the simulative I/R-induced upregulation of TNF-α, ICAM-1 and CXCL16.

Infiltration of neutrophils is a common feature of acute inflammatory reactions. It has been well established that infiltrated leukocytes are important mediators of cardiac I/R injury [12]–[13]. Various approaches used to inhibit neutrophils after myocardial I/R, such as depletion of neutrophils by specific filters and inhibition of adhesion molecules by selective antibodies, can reduce I/R damage [14]. Accumulation of activated neutrophils in I/R myocardium contributes to NR both by physically occluding microvessels as shown by Engler et al. [15] in dogs and by acting as a source of oxygen radicals which lead to severe cell damages and myocardial NR. In this study, we have provided direct evidence for the existence of neutrophil accumulation in the NR area of rabbit I/R myocardium. We verified that these accumulated cells are positive for p65 and express a high level of MPO. In addition, the amount and function of neutrophils in the NR area are significantly greater than that in N and AAR areas, as indicated by MPO levels. These results clearly reflect a higher level of inflammation in the NR area than in N and AAR areas. PDTC markedly suppressed the accumulation of neutrophils in the NR area of the heart, which suggest that NF-κB likely mediates this prominent inflammation. Frantz et al. reported that deletion of NF-κB subunit p50 reduces I/R injury, which is associated with less neutrophil infiltration [16]. Consistently, in our study, application of PDTC reduces the amount of the infiltrated neutrophils in the NR area along with a decrease in the degree of NR, which further suggests that NF-κB is, in fact, involved the mechanism of I/R-induced NR by aggregating inflammation characteristic of neutrolphil accumulation.

Another possible mechanism for neutrophils to promote NR is to adhere to and cross endothelia and clog capillaries [17]. Chemotactic agents and adhesion molecules play essential roles in inflammation by promoting the accumulation of activated neutrophils. Cytokine TNF-α is known for their potent ability to attract leukocytes to inflammatory sites. Our results showed an increase in TNF-α serum protein expression after I/R, which is consistent with the observation made by Gao et al [18]. Adhesion molecule ICAM-1 is a primary determinant of polymorphonuclear neutrophil recruitment by facilitating transmigration of leukocytes across vascular endothelia to exert inflammatory effects. Studies carried out in ICAM-1 deficient mice (*Icam1^−/−^*) [19] or animals receiving antibodies against ICAM-1 [20] have clearly demonstrated a reduction in myocardial necrosis and neutrophils infiltration after I/R. Consistently, serum levels of ICAM-1 in rabbits were induced after the initiation of ischemia and uproar after reperfusion in our study. Intriguingly, we found CXCL16, a small cytokine belonging to the CXC chemokine family, reacted to myocardial I/R even more quickly than TNF-α and ICAM-1 and peaked right after the initiation of reperfusion. CXCL16 is a trans-membrane cytokine that exists in both a membrane bound and soluble form. Membrane bound CXCL16 can not only act as a scavenger receptor for oxidized low-density lipoproteins to facilitate cell adhesion [21] but also can act as an adhesion molecule for leukocytes expressing CXCR6, the sole receptor for CXCL16. More importantly, surface expressed CXCL16 can be cleaved by proteases of the ADAM family to release as a soluble form [22] and thereby serves as a chemo-attractant for CXCR6+ cells. Consequently, the shed chemokine domain of CXCL16 simply forms of a chemotactic gradient for leukocytes expressing CXCR6 to recruit neutrophils [23]. Accumulating evidences have shown that CXCL16 is a marker of inflammation, atherosclerosis and acute coronary syndrome in humans [24]. Given the unique properties of CXCL16 and the data from our study, it is reasonable to propose that CXCL16 may be involved in the inflammatory response of NR by directly promoting the migration of leukocytes to NR area, the very site of violent inflammation. The elevation of TNF-α, ICAM-1 and CXCL16 during reperfusion were suppressed by PDTC, which confirms that NF-κB mediates the increase of these proinflammatory cytokines. Meanwhile, application of PDTC tremendously rescued I/R induced cardiac damages as measured by gross NR extent and ultrastructural lesions. NF-κB was known to be involved in myocardial I/R injury based on the fact that employment of IKK [8] and proteasome inhibition [25] or overexpression of inhibitory IKB protein to suppress NF-κB in animals attenuated myocardial I/R injury. Similar clinic benefit was also observed in patients subjected to cardioplegic arrest on cardiopulmonary bypass received antioxidants to suppress NF-κB expression [26]. However, little information is known about the effects of NF-κB blockade on NR. Our study reveals that suppression of NF-κB contributed to amelioration of anatomic NR by decline of the neutrophils infiltration in NR area as well as the expression of proinflammatory factors. Further study such as measuring the change of actual blood perfusion might be needed to confirm the effect of PDTC treatment on NR phenomenon.

NF-κB is a ubiquitous transcription factor that regulates the expression of immediate-early and stress response genes following a variety of stimuli, many of which are implicated in acute inflammatory responses. Activated NF-κB induces upregulation of TNF-α resulting in I/R injury such as the secretion of inflammatory media, decrease of myocardial contractility, and induction of cardiomyocytes apoptosis [27]. It is well known that cardiac I/R injury is one of the main mechanisms of NR. To explore the mechanism of I/R-associated NR *in vitro*, we investigated HUVECs in the well-established simulative I/R model. Our results confirm that I/R is associated with a systemically increased release of inflammatory cytokines regulated by NF-κB. Indeed, analogous to the findings in rabbit NR model, we observed the increase in TNF-α, ICAM-1 and CXCL16 mRNA and protein levels in HUVECs after simulative I/R. Furthermore, suppression of NF-κB by administration of PDTC or p65 siRNA prior to simulative I/R treatment significantly attenuated the expression of TNF-α, ICAM-1 and CXCL16. Of great interest was CXCL16 mRNA expression that was also stimulated by I/R and associated with an accumulation of the secreted CXCL16 protein in the cell culture supernatants, which suggest a role of this cytokine in the inflammatory process. These results are in agreement with a previous study in which stimulation of endothelial cells with TNF-α and IFN-γ leads to the induction of CXCL16 expression [22]. Because TNF-α is induced by NF-κB very quickly following I/R and therefore may be assumed to function as one of the initiators of a cytokine cascade and dominate the upregulation of CXCL16. Surprisingly, we observed that serum CXCL16 responded to I/R more quickly to I/R than TNF-α. Whether the decrease in CXCL16 level upon suppression of NF-κB is a direct or indirect effect remains to be elucidated. Further investigations are needed to pinpoint the precise mechanism underlying CXCL16 regulation and provide more insights into the role of this novel chemokine in inflammation associated NR. In addition, not only the mRNA and protein levels of NF-κB p65 but also its activity as indicated by nuclear translocation and phosphorylation at Ser536 was promoted by I/R in our study. It is known that IKK phosphorylation to free p65 is one of the mechanisms for p65 activation. In this case, we can not yet reach the conclusion that I/R-induced increase of p65 mRNA/protein level is the only mechanism for the upregulation of TNF-α/ICAM-1/CXCL16.

In summary, by promoting the expression of proinflammatory cytokines TNF-α, ICAM-1 and CXCL16 as well as recruiting neutrophils, NF-κB aggravates inflammatory response and contributes to NR in the setting of myocardial I/R. These findings provide a better understanding of the effects of NF-κB on inflammatory response of I/R-associated NR in the heart. Therefore, inhibition of NF-κB might be a promising approach to prevent NR in patient with AMI underwent reperfusion.
